# Metabolic Phenotyping and Strain Characterisation of *Pseudomonas aeruginosa* Isolates from Cystic Fibrosis Patients Using Rapid Evaporative Ionisation Mass Spectrometry

**DOI:** 10.1038/s41598-018-28665-7

**Published:** 2018-07-19

**Authors:** Emmanuelle E. Bardin, Simon J. S. Cameron, Alvaro Perdones-Montero, Kate Hardiman, Frances Bolt, Eric W. F. W. Alton, Andrew Bush, Jane C. Davies, Zoltan Takáts

**Affiliations:** 1 Department of Surgery and Cancer, Imperial College London, London, United Kingdom; 2 National Heart and Lung Institute, Imperial College London, London, United Kingdom; 3 Department of Paediatric Respiratory Medicine, Royal Brompton and Harefield NHS Foundation Trust, London, United Kingdom

## Abstract

Rapid evaporative ionisation mass spectrometry (REIMS) is a novel technique for the real-time analysis of biological material. It works by conducting an electrical current through a sample, causing it to rapidly heat and evaporate, with the analyte containing vapour channelled to a mass spectrometer. It was used to characterise the metabolome of 45 *Pseudomonas aeruginosa* (*P*. *aeruginosa*) isolates from cystic fibrosis (CF) patients and compared to 80 non-CF *P*. *aeruginosa*. Phospholipids gave the highest signal intensity; 17 rhamnolipids and 18 quorum sensing molecules were detected, demonstrating that REIMS has potential for the study of virulence-related metabolites. *P*. *aeruginosa* isolates obtained from respiratory samples showed a higher diversity, which was attributed to the chronic nature of most respiratory infections. The analytical sensitivity of REIMS allowed the detection of a metabolome that could be used to classify individual *P*. *aeruginosa* isolates after repeated culturing with 81% accuracy, and an average 83% concordance with multilocus sequence typing. This study underpins the capacities of REIMS as a tool with clinical applications, such as metabolic phenotyping of the important CF pathogen *P*. *aeruginosa*, and highlights the potential of metabolic fingerprinting for fine scale characterisation at a sub-species level.

## Introduction

*Pseudomonas aeruginosa* is a ubiquitous, gram-negative bacterium which is readily eliminated by a healthy immune system. However, it can cause both acute and chronic infections, with substantial morbidity and mortality, in a number of clinical scenarios: acute in intubated patients, those with burns, cancer, immunosuppression, and chronic in cystic fibrosis (CF) and other forms of chronic suppurative lung disease^1^. Indeed, up to 60% of adult CF patients are chronically infected with *P*. *aeruginosa* which is associated with a poorer prognosis^2^. The genome of *P*. *aeruginosa* is one of the largest in the bacterial kingdom^3^, 10% of it being dedicated to environmental adaptation^4^. Virulence and persistence-associated mechanisms include a complex quorum sensing communication system which controls the transition to the more resistant biofilm mode of growth^5^. Amongst numerous varieties of secreted toxins, the bio-surfactant rhamnolipids play a multilevel role, facilitating access to nutrients and exhibiting adverse effects on host immune cells or microbial competitors^6^. *P*. *aeruginosa* is thus equipped with a wide range of adaptive responses through versatile metabolic strategies, allowing it to adjust to varying nutrient and oxygen availability, host immune factors and infecting competitors. This makes it particularly suited to the environmental niche of the CF airway^7^.

During the last decade, MS has established itself in clinical laboratories as a quick, reliable, and low-cost method for microbial identification^8,9^. Currently, the main MS platforms which have been introduced use matrix assisted laser desorption ionisation (MALDI) coupled to a time-of-flight (ToF) mass spectrometer. The analysis is conducted on pure bacterial cells embedded in a UV-absorbent matrix which aids ionisation. Microbial identification is based upon the detection of protein fingerprints, mainly of ribosomal origin^10^. Clinical platforms such as the Microflex LT (Bruker Daltonics) and the Vitek MS (bioMerieux) are widely available and MALDI-ToF is now recognised as a standard method for the identification of microorganisms. Whilst these systems accurately classify *P*. *aeruginosa*, other genera prove problematic and overall species level accuracy is 80–90%^9^. Besides, the embedding matrix required for MALDI analysis substantially limits its ability to study lower molecular weight compounds because of the presence of high matrix signal below the mass over charge ratio (*m/z*) 1000. Quorum sensing molecules (QSMs)^11^ and rhamnolipids^12^ have previously been detected using MALDI-ToF, but these methods required extraction from bacterial cultures and a chemical derivatisation or labelling step.

In clinical microbiology, strain level identification is important in understanding epidemiology, population structure, molecular evolution, and in the control of nosocomial and cross-infections^13^. The main methods for detecting high-risk strains rely on molecular typing of DNA, including pulsed-field gel electrophoresis (PFGE), variable-number tandem repeats (VNTR), multilocus sequence typing (MLST), and whole genome sequencing (WGS)^14^. MLST is based upon the DNA sequencing of multiple, highly conserved, housekeeping genes^15^. Currently, the commercially available MS platforms cannot provide strain level classification of microbial species. Nevertheless, MALDI-MS has shown some potential when performed on bacterial protein extracts. To some extent, MALDI-ToF achieved the differentiation of outbreak strains of *Acinetobacter baumannii*^16^, *Staphylococcus aureus* lineages^17^, and five *P*. *aeruginosa* high-risk clones^18^. Fleurbaaij *et al*. recently showed that the ultrahigh resolution and increased dynamic range obtained with a Fourier transform ion cyclotron resonance detector allowed the classification of strains of *P*. *aeruginosa* in accordance with cluster analysis based on amplification fragment length polymorphism (AFLP), which was not achieved with a ToF detector^19^. Unfortunately, this technology remains excessively expensive and cumbersome, making it unsuitable for clinical implementation. Typing methods typically detect changes in slowly evolving regions of the genome however, finer scale characterisation may be a useful adjunct when investigating rapidly evolving characteristics such as virulence determinants.

Rapid evaporative ionisation mass spectrometry (REIMS) is an ambient ionisation MS technique which allows the real-time analysis of biological material. Originally developed as an intra-operative, *in situ* method for tumour margin detection^20^, it has recently been adapted to the study of microbial isolates cultured on agar plates^21^. This method has previously been shown to accurately classify clinically important yeast and bacteria to species level^21–23^. REIMS operates by subjecting microbial biomass grown on a standard agar plate to electric current using electrosurgical bipolar forceps. This causes the biomass to rapidly heat, resulting in the evaporation of particles containing ionised metabolites and structural lipids. The gas phase particles are then introduced into the mass spectrometer using the instrument’s vacuum system^21,23^. Typically, the REIMS spectrum of microorganisms comprises a wide range of structural lipids, originating from cell membranes^21,24^. The phospholipid profile detected in the 600 to 900 *m/z* range provides robust, accurate classification to Gram, genus, and species level^21,23^.

Unlike other MS methods, REIMS provides valuable metabolic information without sample extraction. Here we have explored the potential of REIMS for the direct analysis of *P*. *aeruginosa* cultures. We evaluated *P*. *aeruginosa* intra-species metabolic diversity and looked for virulence-associated metabolites. Additionally, we tested the capacities of REIMS for fine scale *P*. *aeruginosa* characterisation at a sub-species level.

## Results

### Identification of Virulence-Associated Metabolites in REIMS Spectra

**Figure 1:**
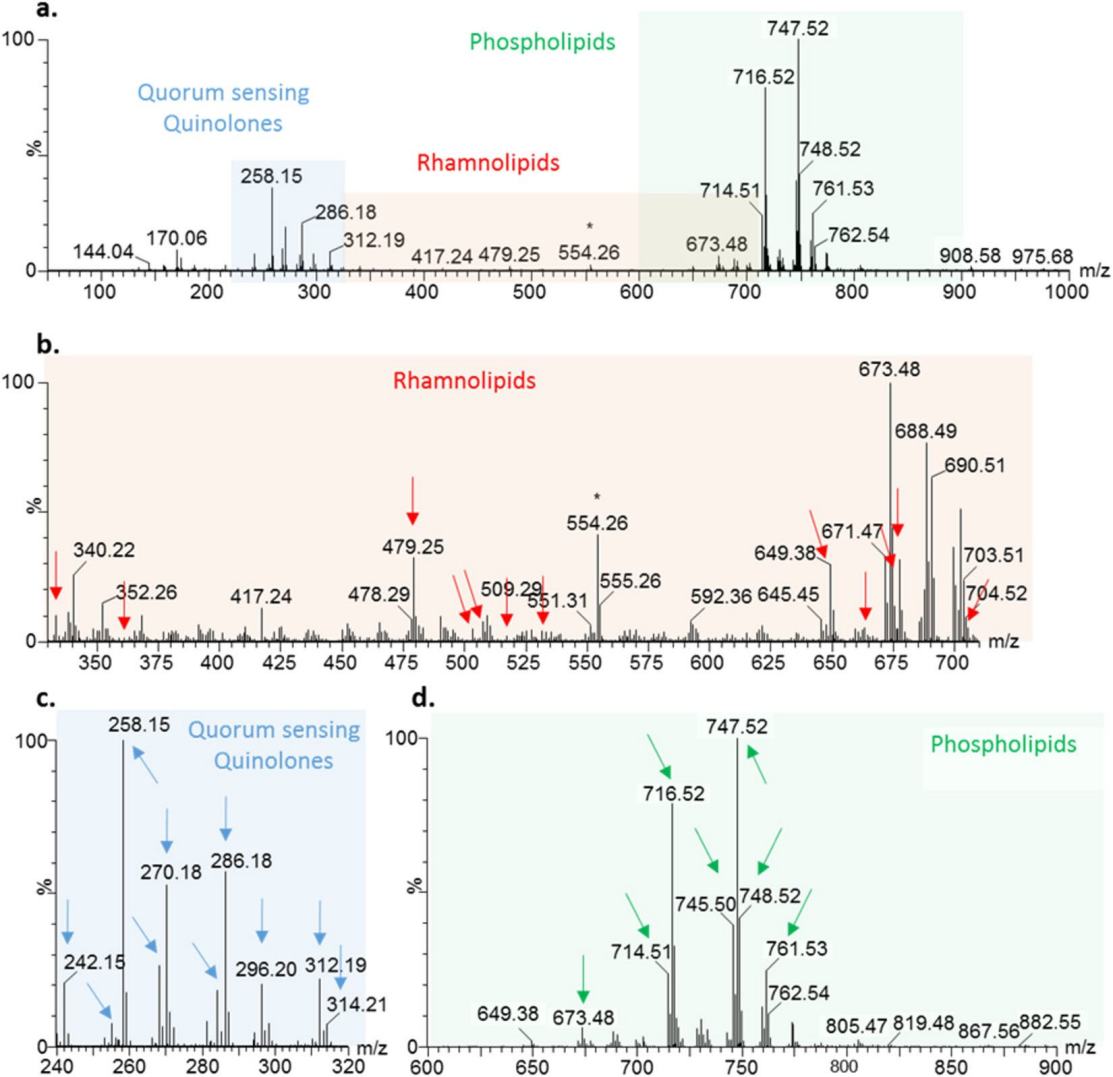
Typical mass spectrum obtained from *P*. *aeruginosa* isolates using REIMS. Acquired using REIMS in negative ion detection mode on CF_01, showed after background removal and mass drift correction. Highlighted spectral regions include (**a**) the 50–1000 *m/z* range showing the most intense features; (**b**) the rhamnolipid *m/z* range of 330–710; (**c**) the QSM *m/z* range of 240–320; and (**d**) the phospholipid *m/z* range 600–900. Arrows highlight some of the annotated spectral features that were determined based on MSMS spectral data.

A typical mass spectrum obtained when applying REIMS on *P*. *aeruginosa* colonies is presented in Fig. 1, after recalibration and background subtraction. The most intense peaks were phospholipids, detected in the 600–900 *m/z* range, deriving mostly from phosphatidyletanolamines (PE) and phosphatidylglycerols (PG). Amongst the most intense phospholipid features, PA(18:1/16:0), PE(18:1/16:1), PE(18:1/16:0), PG(18:1/16:1), PG(18:1/16:0), PG(19:1/16:0), PG(18:1/17:0), PG(18:0/17:1), PG(18:1/18:1), PG(19:1/17:1) were identified using tandem MS (Fig. 1d, Supplementary Table S1).

Twelve *m/z* values, with high intensities in the *m/z* range 240–320, were identified as QSMs, including acylated quinolines and quinolones (or their structural isomer quinolines N-oxide) based on their fragmentation pattern (Fig. 1c, Supplementary Table S1). Both chemical families comprise a series of alkyl chain substitution of seven, nine or eleven carbons, with and without unsaturation. Together with acylated homoserine lactones, they constitute a complex interconnected and self-regulated system that coordinates gene expression as *P*. *aeruginosa* infection progresses^25^. This eventually leads to a decrease in motility and virulence, with establishment of antibiotic, and host-defence resistant populations. Quinolones play an important role in regulating virulence factor production such as elastase, pyocyanin and LecA lectin^25^. The Pseudomonas Quinolone Signal (PQS or 2-heptyl-3-hydroxy-4-quinolone), mixed with 2-heptyl-4-hydroxyquinoline n-oxide (HQNO), usually presented the most intense peak at *m/z* = 258.1472. PQS is produced during the logarithmic phase of growth^26^, and levels in the airways of infected CF patients have been shown to correlate with infection stage^27^.

Likewise, a total of 17 rhamnolipids were identified in the *m/z* range 330–710, as shown in mass spectral annotations in Fig. 1b (fragmentation data shown in Supplementary Table S1). These metabolites are composed of one or two rhamnose sugars, substituted with up to three acyl chains, which accord them amphiphilic properties. Rhamnolipids have various physiological functions which have only partially been elucidated and are known to contribute to the development, adaptability, and persistence of the producing-pathogen. They facilitate access to hydrophobic linear alkanes, which represents a good substrate for *P*. *aeruginosa* growth in harsh environments. Rhamnolipids trigger inflammatory reactions in the lungs and inhibit ciliary function in the host^6^. They have been shown to be required for chronic infection to become established^28,29^, and high levels correlate with clinical decline in CF patients^29^. Additionally, they exhibit antimicrobial activity against a wide range of bacteria and fungi; potentially aiding *P*. *aeruginosa’s* dominance in the adult CF airway. Rhamnolipids also make a noteworthy contribution to PQS activity by enhancing the solubility of PQS in aqueous solutions, thus easing its diffusion from cell to cell^30^. They are involved in swarming motility, and biofilm development by regulating cell-surface physicochemistry, and further cell detachment and dispersion^31^. The overall surfactant properties are determined by the composition of the rhamnolipid profile which relies on strain variations and carbon source availability^32^. Rha-C10-C10 and Rha-Rha-C10-C10 are usually reported to be the most abundant compounds^32^. Yet, within the present population of *P*. *aeruginosa* isolates, the di-rhamnolipids Rha-Rha-C10, Rha-Rha-C10-C10, and Rha-Rha-C10-C12 appeared to be highly prevalent. This indicates disparity in the expressed DNA of our sample set, and/or differences in the growing environment and available nutrients, in comparison to the previous studies.

### REIMS Analysis Shows Higher Metabolic Diversity in *P*. *aeruginosa* Isolated from Airways than those from Non-Respiratory Tract

**Figure 2:**
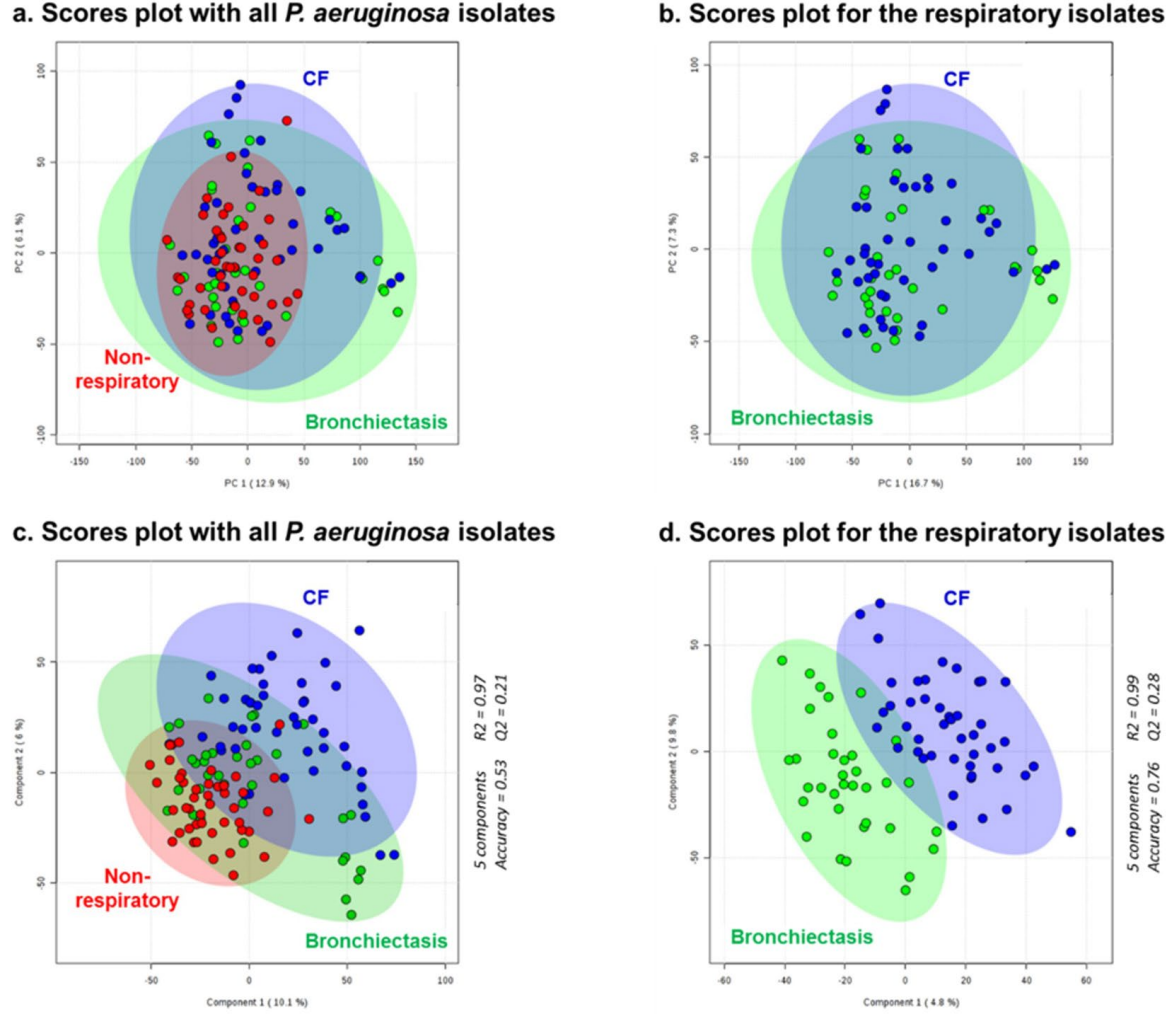
Statistical Analysis of *P*. *aeruginosa* Isolates from CF and Non-CF Patients. Statistical analyses were completed on the 50 to 1100 *m/z* range, after background subtraction and mass drift correction, using the MetaboAnalyst 3.0 platform. The plots all represent components one and two. Shaded areas show 95% confidence intervals of the sample groups. PCA suggests a lower metabolic diversity for the non-respiratory *P*. *aeruginosa* isolates (in red) in (**a**), but does not allow to separate the isolates collected from CF (in blue) from the bronchiectasis (in green) in (**b**). Partial least squares discriminant analysis confirms the lower metabolic diversity for non-respiratory isolates in (**c**) and further shows a separation between isolates collected from CF patients and bronchiectasis patients. The cross-validation details of the PLS-DA are given on the right-hand side for five (**c**) and four (**d**) components; Q2 were above 0.2.

Metabolic profiles of *P*. *aeruginosa* collected from CF patient samples were compared to *P*. *aeruginosa* isolated from non-CF patients which included respiratory samples of patients with bronchiectasis and non-respiratory samples (originating from wounds, blood cultures, urine, drain fluid, ear, vaginal, perineum, rectum, and skin swab samples). Given the high number of features detected with REIMS for every single analysis, it is necessary to employ statistical tools to reduce the dimensionality of the data in order to allow interpretation. Principal component analysis (PCA) is an unsupervised procedure commonly used to condense the information within a dataset into a reduced number of principal components which allow to visualise the highest variance of the results. Partial least square-discriminant analysis (PLS-DA) on the other hand is a supervised method. Whereas unsupervised PCA summarizes the information in an impartial manner, supervised PLS-DA seeks to represent the data according to the variables which allow best the separation of the classes of interest. PCA and PLS-DA plots in Fig. 2 display the area of the 95% confidence interval shading for the various groups of *P*. *aeruginosa* isolates. Although the areas overlap, isolates from the airways of CF and bronchiectasis patients showed substantially higher metabolic diversity compared to those collected from non-respiratory tract samples in Fig. 2a,c. This phenomenon was attributed to the more extended adaptation of the pathogen to its evolving environment, i.e. the lungs, in the case of respiratory infections which are more likely to be chronic. Further separation between the isolates collected from CF or bronchiectasis patients was not apparent using PCA (Fig. 2b) but was made visible by applying PLS-DA, which allows to select the statistical components that are most appropriate to our group separation (Fig. 2d). The overlap observed between the CF and the bronchiectasis isolates in Fig. 2d could not be explained by similarities in mucoid nor any obvious phenotypic characteristics. The separation seemed to be driven mainly by phospholipids as shown when performing HCA using the 50 most differentiating mass spectral bins (Supplementary Fig. S1a and S1b). These bins were mainly comprised within the 600–900 *m/z* range which is typical for phospholipids. They allowed a relative separation between CF from non-respiratory isolates (Supplementary Fig. S1a); and CF from bronchiectasis isolates (Supplementary Fig. S1b). This suggests that the differentiation of the isolates involves structural modifications on the microbial membrane to adapt to its environment. REIMS shows the analytical sensitivity to detect, from microbial isolates grown on culture plates, metabolic diversity induced by previous environmental adaptation. The observation of a higher metabolic diversity within *P*. *aeruginosa* isolates collected from CF patients compared to non-respiratory infections is in line with the phenotypic and biochemical adaptability provided by the large genome of the micro-organism^33^.

### Metabolic Characterisation and Differentiation of *P*. *aeruginosa* CF Isolates using REIMS Analysis

**Figure 3:**
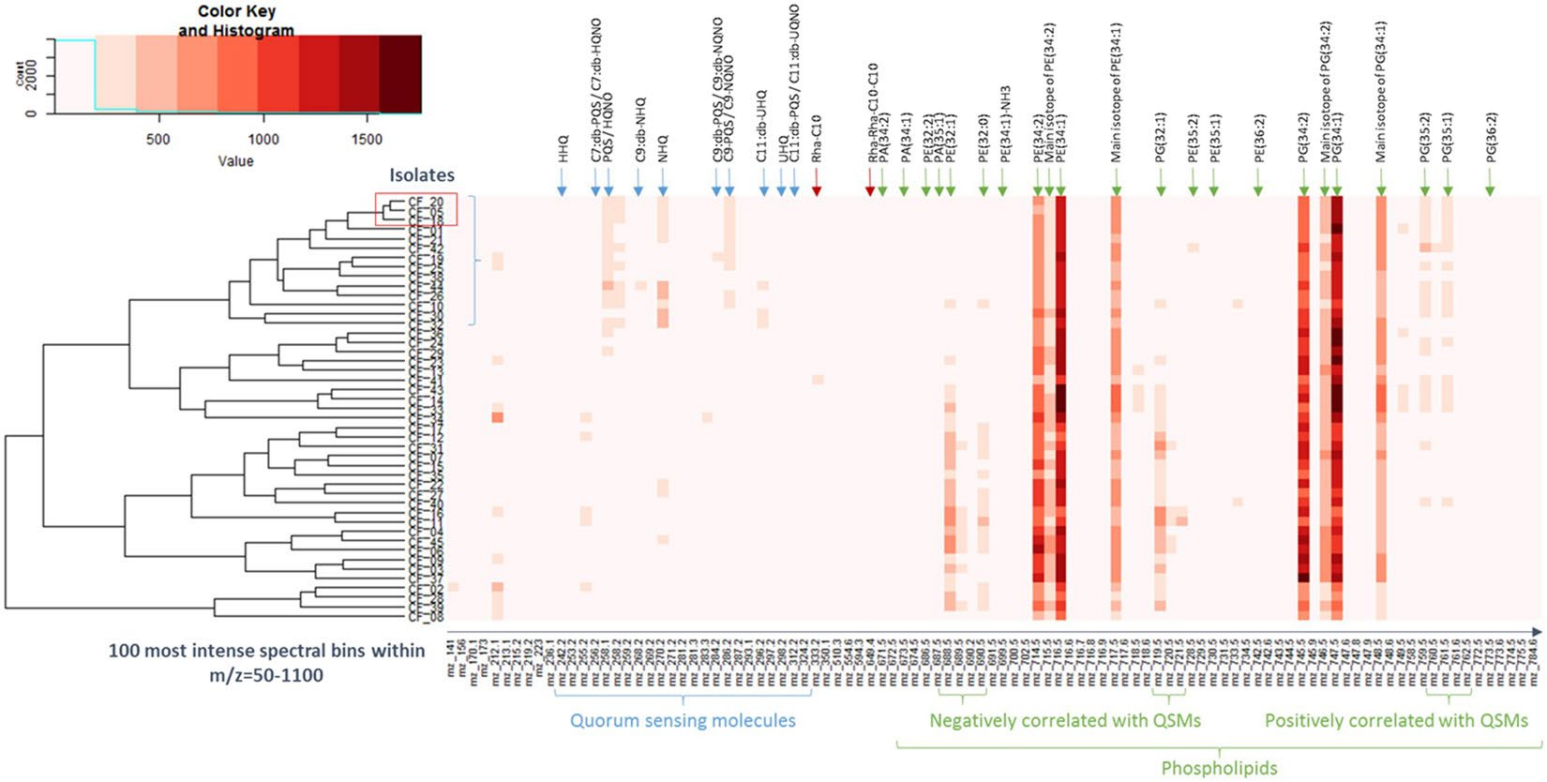
Hierarchical Cluster Analysis using the Metabolic Profiles of 45 *P*. *aeruginosa* Isolates. HCA and associated heat map showing the clustering of 45 *P*. *aeruginosa* isolated from CF lungs, built using the top 100 most intense mass spectral features within the 50–1100 *m/z* range, and spectra averaged on ten replicates for each isolate. Column annotations depict identified metabolic features.

A total of ten analytical repeats were acquired on different days for each of the 45 *P*. *aeruginosa* isolates collected from CF patients. The normalised spectral data, after lock mass correction and background removal, were averaged using the arithmetic mean to produce one spectrum per isolate. HCA was performed using the 100 most intense mean bins in the 50–1100 *m/z* range, which consisted predominantly of QSMs and phospholipids. The resulting dendogram and heat map (Fig. 3) reflect the importance of phospholipids in the metabolic profile, with PE(34:2), PE(34:1), PG(34:2), and PG(34:1) having the highest signal intensity. Isolates with high levels of QSMs formed a subgroup. Interestingly, this subgroup appeared to show a positive correlations with PG(35:2) and PG(35:1) lipids, but a negative correlation with PE(32:1), PE(32:0) and PG(32:1) lipids. This observation may be as a result of QSMs-induced structural modifications to the phospholipid cell membrane and further study may elucidate associated pathways. The clustering also showed that QSMs played an important role in differentiating the *P*. *aeruginosa* isolates.

**Figure 4:**
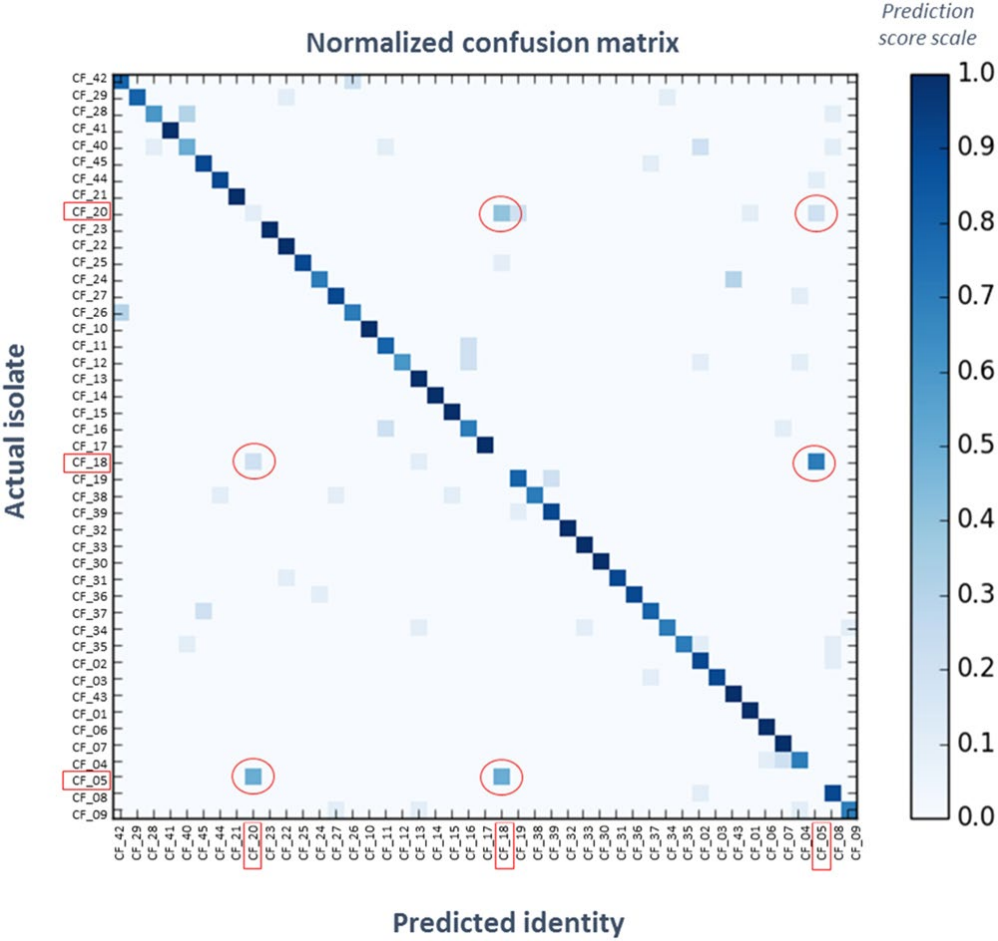
Confusion Matrix for Random Forest Classification of 45 *P*. *aeruginosa* Isolates. Obtained with a set of 45 *P*. *aeruginosa* isolated from CF lungs, using the whole *m/z* range of 10 replicates mass spectra acquired using REIMS. Real and predicted identities are displayed respectively on the left-hand and the bottom sides. The frequency of prediction for each isolate is represented in darkening blue. The isolates circled in red (CF_05, CF_18, CF_20) were predicted as one another. The overall classification using this method was 81% accurate.

The Random Forest machine learning algorithm is a machine learning method used for modelling and classification. It was applied to the *P*. *aeruginosa* dataset comprising ten mass spectra replicates per isolate to construct a prediction model, identifying mass bins that possess the differential capacity to separate between individual isolates. The resulting model was tested using a one-leave-out cross-validation. In this procedure, one replicate is removed from the dataset which is then used to build the prediction model; the prediction model is challenged by classifying the removed replicate. The operation is repeated for every replicate, and predictions are reported in a table called confusion matrix (e.g. Fig. 4): actual classes or identities are displayed on the left-hand side, predicted identities at the bottom, and the frequency of prediction for each class as another is shown as a darkening blue, on a scale from zero to one. If the prediction model is efficient, the replicates are predicted as belonging to their actual class, and the confusion matrix displays a dark blue diagonal. The corresponding F1 scores were calculated for each isolate, and are given in Supplementary Table S3. The mean F1 scores of all 45 isolates represents the overall accuracy of the classification model and was 0.81 – with the maximum value of any model being 1. Of the 45 isolates, only three (7%) had a F1 score below 0.5. However, 27 isolates (60%) possessed a F1 score above the mean of 0.81, and more than a third of the isolates were predicted with a minimum of 90% accuracy. Interestingly, the three isolates (CF_05, CF_18, and CF_20) which possessed the lowest F1 scores, and were unable to be differentiated using the Random Forest algorithm, clustered together on the HCA (Fig. 3); indicating that their metabolic profile was very similar.

This suggests that REIMS has the ability to differentiate between isolates of *P*. *aeruginosa* and to detect changes in the metabolism of the bacteria which may have utility as a tool for monitoring the evolution of an infection.

### Classification of *P*. *aeruginosa* CF Isolates using REIMS Metabolic Fingerprints according to MLST Types

**Figure 5:**
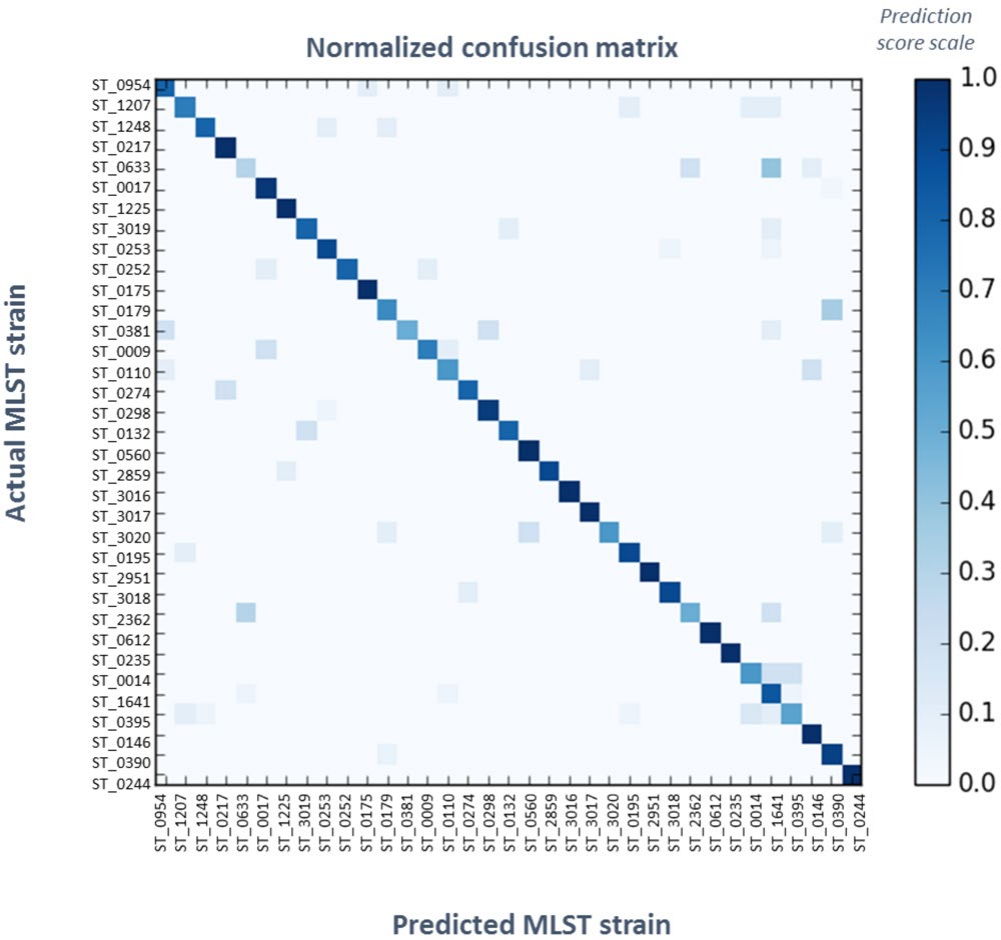
Confusion Matrix for Random Forest Classification of 35 *P*. *aeruginosa* MLST Strains. One-leave-out cross-validation using Random Forest algorithm on a set of 35 strains, grouping 45 *P*. *aeruginosa* isolated from CF lungs with 10 replicates per isolate, using the whole *m/z* range mass spectra acquired using REIMS. Real and predicted identities are displayed respectively on the left-hand and the bottom sides. The frequency of prediction for each isolate is represented in darkening blue. The classification using this method was 83% accurate.

To assess the utility of REIMS for high resolution strain level characterisation, 45 isolates were typed by MLST. Whilst MLST typing methods differ from REIMS as it provides information on slowly evolving genomic regions, the data allows a better understanding of the genetic relationships within the isolates examined. This phylogenetic ‘backbone’ allows us to then explore the additional information offered by REIMS as a finer scale characterisation tool. Of the 45 *P*. *aeruginosa* isolates collected from CF patients, a total of 35 different MLST types were identified; four of them were previously reported as high-risk clones of *P*. *aeruginosa*^18^ (ST_0175, ST_0235, ST_0253, ST_0395), and eight types had at least two isolates (Supplementary Table S4). The previously poorly predicted isolates (CF_05, CF_18, and CF_20) were shown to belong to the same strain – namely ST_0390. In order to evaluate the capacity of REIMS analysis to provide sub-species information and differentiation, the ten replicates for each isolate of each strain were grouped, and tested for classification according to MLST types. The confusion matrix for leave-one-out cross-validation obtained with the Random Forest model is shown in Fig. 5, and the individual F1 scores, precision, and recall scores shown in Supplementary Table S4. Even though REIMS offers a more rapidly evolving picture of *P*. *aeruginosa* strains, on average, isolates were classified with 83% concordance with MLST types. This indicates that metabolomic profiling offers some level of stable strain characterisation. Notably, it also provides insights into more rapidly evolving targets including QSMs. Only one isolate (CF_16) was predicted below 0.5 in accordance with its type (ST_0633), 60% of the replicates were accurately predicted above the 0.83 average, and 40% were above 0.9. In the case of ST_0179 and ST_0395, MS data revealed differences in the quorum sensing and phospholipid regions respectively, thereby highlighting the higher resolution of the information provided by REIMS. In the case of ST_0633, and other poorly predicted types with single isolates, the low classification scores are likely to be due to insufficient reproducibility, especially in the case of mucoid strains.

## Discussion

*P*. *aeruginosa* isolates from respiratory samples displayed a substantially higher diversity in their metabolic profile, suggesting that REIMS is able to detect subsequent changes in isolates exposed to different conditions. It also allowed the detection and identification of lower mass metabolites, including virulence-associated QSMs and rhamnolipids. These are key factors for an infection to progress to chronicity, yet the study of this type of metabolites is limited by the performance, the cost, and the complexity of available technologies. The routine detection of these metabolites is not possible with the current clinical MALDI-ToF platforms, and REIMS could be useful in the assessment of pathogen virulence, potentially assisting in risk stratification.

In addition, REIMS classified strains with 83% concordance to MLST types, suggesting that metabolic fingerprinting can provide some strain level information. Indeed, the Random Forest model utilised the whole range of *m/z*, including phospholipids as well as low molecular weight metabolites, such as QSMs and rhamnolipids, which have been shown to vary between strains of *P*. *aeruginosa*^34^. Some isolates grouped under the same type showed a similar metabolome, whereas others displayed spectral differences, particularly in the quorum sensing and phospholipid ranges. Whilst traditional typing tools, such as MLST and VNTR, rely on slowly evolving genomic regions, REIMS could be used as an adjunct to provide fine scale classification, based on more rapidly evolving metabolic expression. It has been suggested that ‘resistance is often concentrated in a few strains’ which are more frequently observed within hospital isolates^35^. These strains can be found worldwide and have been dubbed ‘high- risk clones’^36^. Further research is needed to demonstrate if REIMS can be used to identify virulence-associated variations which would allow the rapid identification of such high-risk clones.

It is further worth noting that culturing conditions have previously been reported to influence the detected metabolic profiles, mostly in terms of intensities^21^. The population structure at the time of analysis, i.e. whether the bacteria were growing as single colonies or as a lawn, also impacted signal intensities in the quorum sensing and rhamnolipid ranges (unpublished data). Therefore, for the purpose of this study, growing time and medium were standardised, and biomass of each sample was analysed from various sites on the plates to account for the natural metabolic diversity within one isolate. Nevertheless, it will be interesting to investigate these changes more precisely in the future, as REIMS improves our understanding of how *P*. *aeruginosa* evolves and adapts to its environment. Furthermore, the mucoid isolates (CF_2, CF_8, CF_12, CF_28, CF_35, CF_39; Table S2) showed lower reproducibility, with a higher background due to their higher water content, as well as lower intensities of QSMs and different profiles of phospholipids. However, it is not possible at the moment to establish whether this reflects the metabolic expression of the bacteria, or if this is a technical effect due to the different physical nature of the sample. These isolates and associated MLST types tended to be predicted as each other which could explain their slightly lower prediction scores, together with a poorer reproducibility (Figs 4 and 5, Tables S3 and S4).

The introduction of MALDI-ToF MS based platforms have dramatically changed the current clinical workflows within microbiology laboratories. REIMS and MALDI-ToF MS have been shown to speciate micro-organisms with an overall accuracy of *ca*. 90%, although the libraries commercially available for MALDI-ToF encompass more species^9,37^. Nevertheless, other notable differences in the methodology and analytical process impact capacities and applications of the two technologies. MALDI-ToF speciation relies on the profiling of highly conserved ribosomal proteins with high *m/z*. The presence of matrix peaks, together with the poor resolution of the linear ToF, limits the identification of low *m/z* metabolites, or require extensive preliminary extraction. In contrast, REIMS is performed directly onto bacterial biomass and classifies microbes mainly based upon the lower *m/z* phospholipid region. We showed here that lower mass interesting metabolites are also easily detected. REIMS may therefore provide valuable metabolic data and fine scale characterisation information, alongside species-level identification. Whilst many typing methods rely upon slowly evolving regions of the microbial genome, tools such as REIMS may offer insights into more rapidly evolving variations which could, for example, be seen in virulence associated products.

The detected metabolome may be further linked with clinical disease indicators such as the rate of disease progression or pulmonary exacerbations. Based upon the unique MS profile obtained with no sample extraction, it is plausible that the metabolites identified here can be detected in native samples such as sputum, and serve as universal chemical markers for culture-free diagnostic methods, using not only MS techniques^38^ but also alternative methods such as semi-conductor or bio-sensors^39^. This would allow for an earlier detection of *P*. *aeruginosa* acquisition by CF patients, without the requirement for culturing, significantly decreasing the time for diagnosis and allow rapid treatment to be provided.

## Methods

### Collection of Bacterial Isolates

CF bacterial isolates had originally been cultured by the Clinical Microbiology Laboratory of the Royal Brompton Hospital (London, UK) from sputum and cough swabs collected from CF patients attending routine clinical visits. The isolates had been first identified through culture on selective cetrimide agar according to standard laboratory practice. Additional isolates of *P*. *aeruginosa* were collected from non-CF patients: 35 from respiratory samples provided by patients with bronchiectasis attending routine clinical visits at the Royal Brompton Hospital (London UK); 45 from non-respiratory tract samples, as part of routine clinical identification at Charing Cross Hospital (London, UK). These *P*. *aeruginosa* isolates were cultured from samples collected from wounds, blood cultures, urine, drain fluid, and ear, vaginal, perineum, rectum, and skin swabs. After initial collection and culturing, all isolates were stored at −80 °C in CryoVials (Microbank^TM^, Pro-Lab Diagnostics, Bromborough, UK), following manufacturer’s guidelines, until cultured for REIMS analysis.

### Species Level Confirmation of Bacterial Isolate Identification using MALDI-ToF Mass Spectrometry

The identity of all microbial species was verified using the Microflex LT MALDI-ToF instrument (Bruker Daltonics, Coventry, UK) as previously described^23^. In brief, single colonies were placed onto a 24-spot steel plate and embedded in an acidic solution of α-cyano-4-hydroxycinnamic acid as a UV-adsorbent matrix. Samples were analysed after drying following the manufacturer’s instructions. Data were processed using the Bruker Biotyper software (version 3.0) and library (version 5.0) for taxonomic classification of the isolates. The identity of the 135 *P*. *aeruginosa* isolates were confirmed.

### REIMS Analysis of Bacterial Cultures

Prior to REIMS analysis, isolates were cultured on Colombia blood agar (Oxoid, Basingstoke, U.K) for 24 hours at 37 °C in normal aerobic atmosphere. The 45 *P*. *aeruginosa* isolates from CF patients were grown and analysed ten times on different days, six grew mucoid more than once (further details are given in Table S2 in supporting information). This was to evaluate reproducibility, clustering and trends in molecular expression, as well as potential for isolate recognition. REIMS analyses were performed as previously described^23^. Handheld bipolar forceps (Erbe Elektromedizin, Tübingen, Germany) were used as a sampling probe. A biomass covering approximately 2 mm^2^ of one probe was collected directly from the medium. On drawing the probes together an electrical power of 70 W was applied using an electrical surgical generator (ERBE ICC 300, Erbe Elektromedizin) causing the bacterial material to rapidly heat. The resulting aerosol was channelled to a XEVO G2-XS Q-ToF instrument (Waters Corporation, Wilmslow, UK). Prior to the entry of the analyte containing vapour to the MS instrument, it was mixed in a T-piece device with isopropanol (Chromasolv® LC-MS grade, Sigma-Aldrich, St Louis, MO, USA) containing 0.01 g/L of leucine encephalin (VWR, Radnor, Pennsylvania, USA) as a lock mass compound, with a solvent flow rate of 0.2 mL/min. Negative ions were detected in sensitivity mode over the 50 to 2500 *m/z* range, at a scan time of 1 second, with a resolution of 22,000. A minimum of three individual measurements were acquired per isolate analysis.

### Data Processing and Statistical Analysis

Data pre-processing was performed using the Offline Model Builder (version 1.1.29.0) software (Waters Research Centre, Budapest, Hungary) and included combining scan measurements per sample, background subtraction, and correction for mass drift using leucine-enkephalin negative ion *m/z* 554.2516. Resulting spectra were normalised according to total ion count and binned to 0.1 *m/z* bins. The lockmass compound peak and its isotopes (555.2518 and 556.2485) were excluded from further statistical processing. Statistical analysis was carried out afterwards on the Metaboanalyst 3.0 platform^40^. Generalized logarithmic transformation and Pareto scaling (data mean-centring and division by the square root of standard deviation of each variable) were applied on the 5000 most intense median bin values. Principal component analysis (PCA), partial least square – discriminant analysis (PLS-DA) and hierarchical cluster analysis (HCA) were then completed. Supervised classification was performed using the machine learning scikit-learn package^41^ and Random Forest algorithm^42^. The F1 scores – harmonic mean of the precision (the fraction of the prediction that is accurate) and the sensitivity (the fraction of samples that are correctly predicted) – were calculated and used to assess the classification accuracy. Ions of interest were annotated using tandem mass spectrometry and fragment identification.

### Determination of *P*. *aeruginosa* Strain through MLST Typing

The 45 *P*. *aeruginosa* isolates collected from CF patients were cultured on Colombia blood agar (Oxoid) for 24 hours at 37 °C, under normal aerobic conditions. For each isolate, a single colony from the agar culture plate was used to inoculate 10 mL of brain heart infusion broth (Oxoid) and then cultured overnight at 37 °C, under a normal aerobic atmosphere. After this, a 2 mL aliquot of culture media was subjected to centrifugation for three minutes at 21,000 × *g*. The supernatant was removed and the cell pellet used for DNA extraction using a Fast DNA SPIN kit (MP Biomedical, Germany), following the manufacturer’s standard instructions. Bead beating was performed in FastPrep 24 instrument (MP Biomedical) using three consecutive cycles of 30 seconds at 6.0 m/sec, each divided by cooling at −20 °C for 3 min. DNA was eluted into 100 µL of PCR grade water (Roche, UK). The success of DNA extractions was determined through visualisation of 5 µL of eluted DNA after running on an 1.0% agarose gel for one hour at 100 volts in 1X tris acetic acid EDTA (TAE) buffer. All isolate extractions displayed an intense band of high molecular weight DNA (>20 kbp). To determine DNA concentration, 1 µL of a one in ten dilution, using PCR grade water (Roche), was used in Qubit fluorometery using the dsDNA Broad sensitivity kit (Invitrogen, UK) and a Qubit 3.0 fluorometer for DNA concentration. The diluted DNA was carried forwarded for whole genome sequencing.

Whole genome sequencing was completed by the MicrobesNG service at the University of Birmingham (Birmingham, UK) using the HiSeq. 2500 platform with 2 × 250 bp sequencing. DNA sequencing libraries were prepared using the NexteraXT kit (Illumina, Cambridge, UK). As part of the MicrobesNG service, the 45 *P*. *aeruginosa* libraries were run alongside 339 other DNA sequencing libraries so that 384 libraries were sequenced on one run of the HiSeq. 2500 platform. Resulting DNA sequences were merged, trimmed for quality, and aligned to the *P*. *aeruginosa* reference genome as part of the MicrobesNG sequence analysis pipeline. From the whole genome sequence assemblies for each isolate, the relevant gene sequences for the seven housekeeping genes used in the MLST scheme^43^ were extracted and the PubMLST website used to complete strain match searches under standard operating parameters. Five novel MLST types were assigned a new ST number through the PubMLST website where the data have been deposited.

### Data Availability

The datasets generated and analysed during the current study are available on the MetaboLights website, study number MTBLS644.

## Electronic supplementary material

Supporting Information
