# Supplementary material for: Metabolic Phenotyping and Strain Characterisation of Pseudomonas aeruginosa Isolates from Cystic Fibrosis Patients Using Rapid Evaporative Ionisation Mass Spectrometry
Source: Sci Rep. 2018 Jul 19;8:10952. doi: 10.1038/s41598-018-28665-7 (PMC6053451; doi:10.1038/s41598-018-28665-7)
Supplement: Supplementary file 1 — Supporting Information [file 41598_2018_28665_MOESM1_ESM.docx]

**Supporting Information**

**Metabolic Phenotyping and Strain Characterisation of *Pseudomonas aeruginosa* Isolates from Cystic Fibrosis Patients Using Rapid Evaporative Ionisation Mass Spectrometry**

Emmanuelle E. Bardin^1^; Simon J. S. Cameron^1^; Alvaro Perdones-Montero^1^; Kate Hardiman^1^; Frances Bolt^1^; Eric W.F.W. Alton^2^; Andrew Bush^2, 3^; Jane C. Davies^2, 3^; Zoltan Takáts^1*^

1. Dept of Surgery and Cancer, Imperial College London, London, United Kingdom.

2. National Heart and Lung Institute, Imperial College London, London, United Kingdom.

3. Department of Paediatric Respiratory Medicine, Royal Brompton and Harefield NHS Foundation Trust, London, United Kingdom.

* Communicating Author: Prof. Zoltan Takáts, z.takats@imperial.ac.uk

**Table S1.** Identification of significant molecules based on MSMS spectral data acquired with direct REIMS analysis on *P. aeruginosa* colonies without preliminary chromatographic separation. Parent ions may therefore represent a mix of molecules. The four most intense fragment ions are given together their parent ion exact mass. Intensities are given related to the most intense fragment ion. Unattributed fragment ions were included in the table; isotope ions were excluded.

| **Quorum Sensing Molecules** | **Theoretical *m/z* [M-H]^-^** | **CID** | **Resolution** | **Four main fragments m/z**  **(intensity relative to the most abundant fragment)** | | | | **Parent ion**  **[M-H]^-^** | **Mass deviation (ppm)** |
| --- | --- | --- | --- | --- | --- | --- | --- | --- | --- |
| C7:db-HHQ | 240.1394 | 26 | 12 | 157.1 (100%) | 170.1 (62%) | 182.1 (38%) | 143.0 (33%) | 240.1357 (37%) | 15 |
| HHQ | 242.1550 | 28 | 12 | 157.1 (100%) | 170.1 (30%) | 143.0 (12%) | 115.0 (7%) | 242.1521 (30%) | 12 |
| C7:db-PQS  C7:db-HQNO | 256.1343 | 29 | 12 | 157.1 (100%) | 170.1 (45%) | 186.1 (41%) | 184.0 (39%) | 256.1312 (32%) | 12 |
| PQS  HQNO | 258.1500 | 29 | 12 | 173.0 (100%) | 170.1 (45%) | 186.1 (28%) | 159.0 (15%) | 258.1472 (34%) | 11 |
| C9:db-NHQ | 268.1707 | 30 | 18 | 157.1 (100%) | 182.1 (89%) | 196.1 (52%) | 143.0 (19%) | 268.1678 (33%) | 11 |
| NHQ | 270.1863 | 30 | 18 | 157.1 (100%) | 170.1 (44%) | 143.0 (16%) | 184.1 (11%) | 270.1834 (35%) | 11 |
| C9:db-PQS  C9:db-NQNO | 284.1656 | 25 | 18 | 196.1 (100%) | 144.0 (37%) | 173.0 (29%) | 158.1 (26%) | 284.1628 (21%) | 10 |
| C9-PQS  C9-NQNO | 286.1813 | 25 | 18 | 170.1 (100%) | 173.0 (47%) | 144.0 (29%) | 186.1 (15%) | 286.1784 (35%) | 10 |
| C11:db-UHQ | 296.2020 | 29 | 18 | 158.1 (100%) | 157.1 (95%) | 170.1 (43%) | 258.2 (27%) | 296.1992 (26%) | 9 |
| UHQ | 298.2176 | 32 | 18 | 157.1 (100%) | 170.1 (56%) | 158.1 (16%) | 143.0 (16%) | 298.2136 (37%) | 14 |
| C11:db-PQS  C11:db-UQNO | 312.1969 | 23 | 18 | 170.1 (100%) | 144.0 (33%) | 173.0 (23%) | 294.2 (18%) | 312.1941 (40%) | 9 |
| C11-PQS  C11-UQNO | 314.2126 | 28 | 18 | 170.1 (100%) | 144.0 (35%) | 173.0 (26%) | 296.0 (15%) | 314.2092 (38%) | 11 |

| **Rhamnolipids** | **Theoretical *m/z* [M-H]^-^** | **CID** | **Resolution** | **Four main fragments m/z**  **(intensity relative to the most abundant fragment)** | | | | **Parent ion**  **[M-H]^-^** | **Mass deviation (ppm)** |
| --- | --- | --- | --- | --- | --- | --- | --- | --- | --- |
| Rha-C10 | 333.1919 | 13 | 18 | 169.1 (100%) | 297.2 (7%) | 163.1 (6%) | 101.0 (6%) | 333.1886 (28%) | 10 |
| Rha-C12 | 361.2232 | 14 | 18 | 197.2 (100%) | 325.2 (21%) | 343.3 (8%) | 151.1 (7%) | 361.2212 (29%) | 5 |
| Rha-Rha-C10 | 479.2498 | 22 | 15-18 | 205.1 (100%) | 143.1 (55%) | 169.1 (50%) | 282.3 (36%) | 479.2483 (29%) | 3 |
| Rha-C10-C10 | 503.3226 | 14 | 18 | 333.2 (100%) | 169.1 (94%) | 339.3 (17%) | 163.1 (7%) | 503.3209 (35%) | 3 |
| Rha-Rha-C12 | 507.2811 | 22 | 15-18 | 205.1 (100%) | 197.2 (63%) | 143.1 (54%) | 131.1 (36%) | 507.2790 (46%) | 4 |
| Rha-C10-C10-CH3  Rha-C11-C9-CH3 | 517.3382 | 16 | 18 | 169.1 (100%) | 333.2 (89%) | 183.1 (66%) | 347.2 (48%) | 517.3353 (39%) | 6 |
| Rha-C10-C12:1 | 529.3382 | 15 | 18 | 333.2 (100%) | 169.1 (79%) | 151.2 (15%) | 365.3 (9%) | 529.3360 (31%) | 4 |
| Rha-C10-C12 | 531.3539 | 15 | 18 | 169.1 (100%) | 333.2 (99%) | 197.2 (48%) | 361.2 (37%) | 531.3518 (37%) | 4 |
| Rha-C10-C14  Rha-C12-C12 | 559.3852 | 16 | 18 | 197.2 (100%) | 361.2 (68%) | 523.4 (21%) | 225.2 (12%) | 559.3815 (31%) | 7 |
| Rha-Rha-C10-C10 | 649.3805 | 19 | 15-18 | 479.3 (100%) | 169.1 (34%) | 205.1 (20%) | 393.4 (15%) | 649.3790 (33%) | 2 |
| Rha-Rha-C10-C10-CH3  Rha-Rha-C11-C9-CH3 | 663.3961 | 22 | 15-18 | 479.3 (100%) | 493.4 (54%) | 205.1 (46%) | 169.1 (45%) | 663.3970 (78%) | -1 |
| Rha-Rha-C10-C12:1 | 675.3961 | 20 | 15-18 | 479.3 (100%) | 393.4 (36%) | 169.1 (26%) | 205.1 (21%) | 675.3952 (37%) | 1 |
| Rha-Rha-C10-C12 | 677.4118 | 22 | 15-18 | 479.3 (100%) | 169.1 (41%) | 205.1 (34%) | 507.4 (33%) | 677.4097 (20%) | 3 |
| Rha-Rha-C12-C12 | 705.4431 | 20 | 15-18 | 507.4 (4%) | - | - | - | 705.4482 (100%) | -7 |
| **Lipids** | **Theoretical *m/z* [M-H]^-^** | **CID** | **Resolution** | **Four main fragments m/z**  **(intensity relative to the most abundant fragment)** | | | | **Parent ion**  **[M-H]^-^** | **Mass deviation (ppm)** |
| LPG(18:1) | 509.2885 | 20 | 18 | 281.3 (100%) | 153.0 (8%) | 227.1 (4%) | - | 509.2871 (34%) | 3 |
| PA(16:1/18:1) | 671.4657 | 22 | 18 | 281.3 (100%) | 253.2 (41%) | 417.3 (40%) | 435.3 (21%) | 671.4642 (37%) | 2 |
| PA(16:0/18:1) | 673.4814 | 22 | 18 | 281.3 (100%) | 255.2 (62%) | 417.3 (37%) | 153.0 (22%) | 673.4791 (34%) | 3 |
| PE(16:1/16:1)  PE(18:1/14:1) | 686.4766 | 22 | 18 | 253.2 (100%) | 281.3 (22%) | 255.2 (17%) | 225.2 (13%) | 686.4785 (29%) | 3 |
| PA(16:0/19:1)  PA(18:1/17:0) | 687.497 | 22 | 18 | 255.2 (100%) | 281.3 (59%) | 295.3 (38%) | 253.2 (38%) | 687.4922 (44%) | 7 |
| PE(16:1/16:0)  PE(18:1/14:0) | 688.4923 | 22 | 18 | 253.2 (100%) | 255.2 (49%) | 227.2 (27%) | 281.3 (23%) | 688.4911 (43%) | 2 |
| PE(16:0/16:0) | 690.5079 | 22 | 18 | 255.2 (100%) | - | - | - | 690.5034 (11%) | 7 |
| PE(18:1/16:0) -NH3 | 699.4970 | 24 | 18 | 255.2 (100%) | 281.3 (63%) | - | - | 699.4946 (44%) | 3 |
| PE(18:1/16:1) | 714.5079 | 24 | 18 | 253.3 (100%) | 281.3 (42%) | 478.4 (9%) | 460.4 (2%) | 714.5057 (34%) | 3 |
| PE(18:1/16:0) | 716.5236 | 24 | 10-18 | 255.3 (100%) | 281.3 (68%) | 478.4 (8%) | - | 716.5201 (47%) | 5 |
| PG(18:1/14:0)  PG(16:1/16:0) | 719.4868 | 28 | 10-18 | 253.3 (100%) | 255.3 (56%) | 281.3 (21%) | 227.2 (17%) | 719.4871 (48%) | 0 |
| PE(17:1/18:1)  PE(16:1/19:1) | 728.5236 | 25 | 10-18 | 267.3 (100%) | 281.3 (43%) | 478.4 (7%) | 253.3 (7%) | 728.5198 (39%) | 5 |
| PE(16:0/19:1)  PE(17:0/18:1)  PE(17:1/18:0) | 730.5392 | 26 | 10-18 | 295.3 (100%) | 255.3 (36%) | 281.3 (23%) | 267.3 (23%) | 730.5308 (44%) | 11 |
| PE(18:1/18:1)  PE(19:1/17:1) | 742.5392 | 25 | 10-18 | 281.3 (100%) | 267.3 (12%) | 295.3 (8%) | 478.4 (7%) | 742.5331 (36%) | 8 |
| PG(18:1/16:1) | 745.5025 | 28 | 10-18 | 253.3 (100%) | 281.3 (45%) | 509.4 (7%) | 153.0 (4%) | 745.4998 (49%) | 4 |
| PG(18:1/16:0) | 747.5181 | 30 | 10-18 | 255.3 (100%) | 281.3 (76%) | 253.3 (10%) | 153.0 (6%) | 747.5148 (42%) | 4 |
| PG(18:1/17:1)  PG(19:1/16:1) | 759.5181 | 35 | 15-17 | 253.2 (100%) | 267.2 (98%) | 281.3 (49%) | 295.3 (43%) | 759.5161 (53%) | 3 |
| PG(19:1/16:0)  PG(18:1/17:0)  PG(18:0/17:1) | 761.5338 | 35 | 15-17 | 295.3 (100%) | 255.2 (88%) | 281.3 (7%) | 267.2 (6%) | 761.5291 (50%) | 6 |
| PG(19:0 / 16:0) | 763.5283 | 38 | 15-17 | 255.2 (100%) | 295.3 (77%) | 297.3 (46%) | 257.3 (32%) | 763.5297 (37%) | 2 |
| PG(18:1/18:1)  PG(19:1/17:1) | 773.5338 | 36 | 15-17 | 281.3 (100%) | 267.2 (59%) | 295.3 (27%) | 153.0 (5%) | 773.5305 (40%) | 4 |
| PC(16:0/18:1) +Cl | 794.5342 | 33 | 15-17 | 779.7 (100%) | 281.3 (51%) | 255.3 (46%) | 253.2 (39%) | 794.5327 (30%) | 2 |
| PC(16:0/18-OH) +Cl | 812.5447 | 37 | 15-17 | 281.3 (100%) | 255.3 (84%) | 253.2 (38%) | 417.3 (31%) | 812.549 (43%) | 5 |
| NAPE(52:2) | 980.7689 | 43 | 15-17 | 255.3 (100%) | 281.3 (67%) | 295.3 (35%) | 267.3 (31%) | 980.7604 (43%) | 9 |

**Figure S1.** Hierarchical cluster analysis and associated heat maps built using the normalised intensities of the top 50 most differentiating mass spectral bins, based on *P* values of univariate *T­*-tests, within the 50 to 1100 *m/z* range. *P. aeruginosa* isolates collected from CF patients are shown in blue, those from bronchiectasis patients in green, and those from non-respiratory tract samples in red. Heat map in (a) shows a relative separation of the CF from the non-respiratory isolates while the bronchiectasis isolates seem homogeneously distributed. Heat map in (b) shows a trend in separating the CF from the bronchiectasis isolates.

**
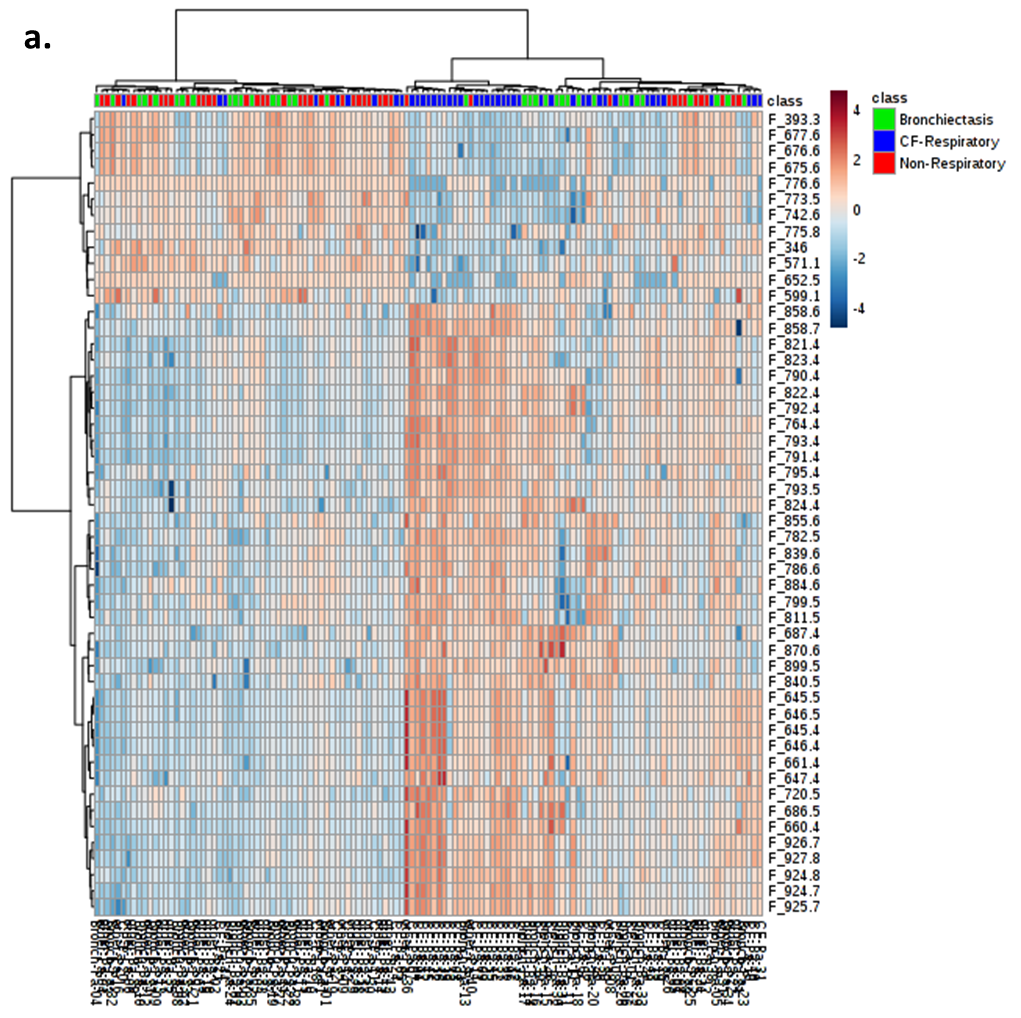
**

**
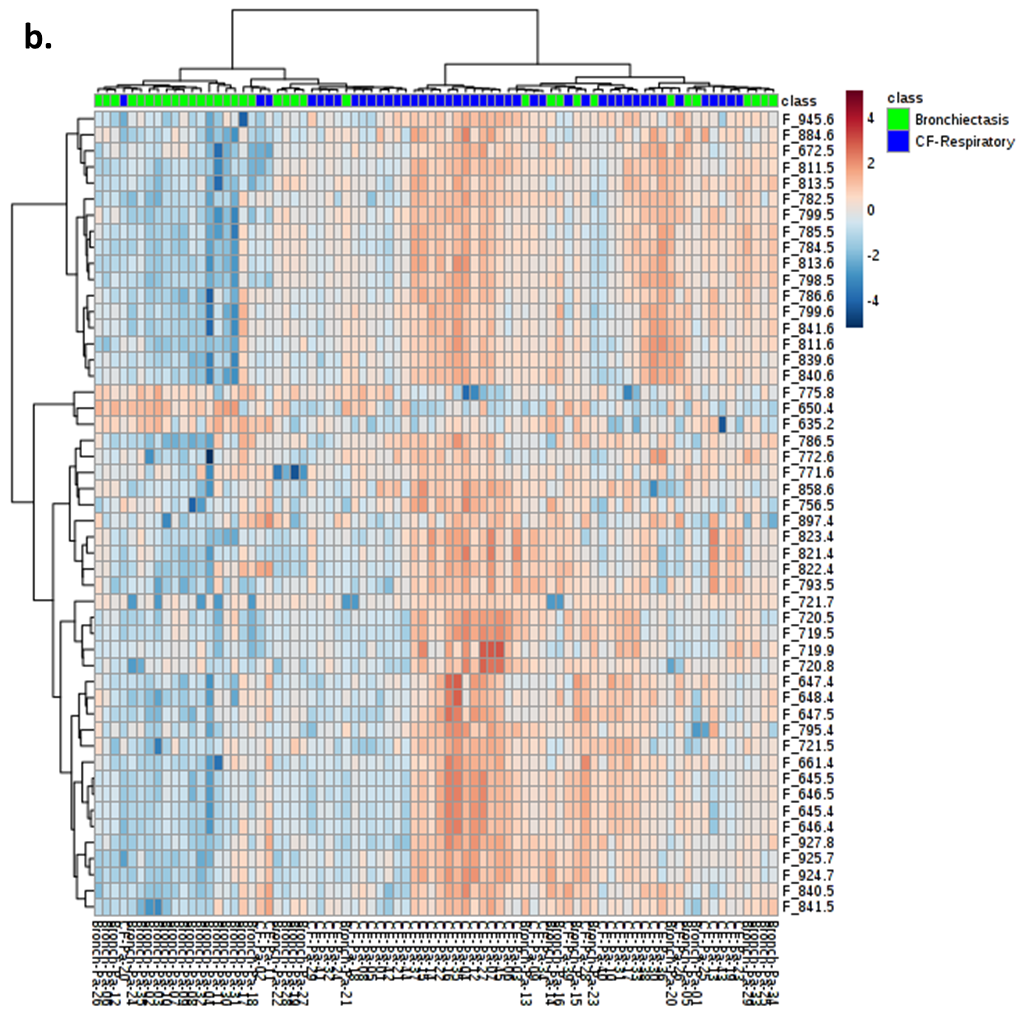
**

**Table S2.** Analysis dates for the 10 replicates of the 45 *P. aeruginosa* isolated from CF lungs. Isolates which grew mucoid more than once are indicated.

| **Isolates** | **Mcoid** | **Day 1** | **Day 2** | **Day 3** | **Day 4** | **Day 5** | **Day 6** | **Day 7** | **Day 8** | **Day 9** | **Day 10** | **TOTAL** |
| --- | --- | --- | --- | --- | --- | --- | --- | --- | --- | --- | --- | --- |
| **CF-01** |  | - | - | 1 | - | 2 | 1 | 1 | 2 | 2 | 1 | 10 |
| **CF-02** | X | - | - | 1 | - | 2 | 1 | 1 | 2 | 2 | 1 | 10 |
| **CF-03** |  | - | - | 1 | - | 2 | 1 | 1 | 2 | 2 | 1 | 10 |
| **CF-04** |  | - | - | 1 | - | 2 | 1 | 1 | 2 | 2 | 1 | 10 |
| **CF-05** |  | - | - | 1 | - | 2 | 1 | 1 | 2 | 2 | 1 | 10 |
| **CF-06** |  | - | - | 1 | - | 2 | 1 | 1 | 2 | 2 | 1 | 10 |
| **CF-07** |  | - | - | 1 | - | 2 | 1 | 1 | 2 | 2 | 1 | 10 |
| **CF-08** | X | - | - | 1 | - | 2 | 1 | 1 | 2 | 2 | 1 | 10 |
| **CF-09** |  | - | - | 1 | - | 2 | 1 | 1 | 2 | 2 | 1 | 10 |
| **CF-10** |  | - | - | 1 | - | 2 | 1 | 1 | 2 | 2 | 1 | 10 |
| **CF-11** |  | - | - | 1 | - | 2 | 1 | 1 | 2 | 2 | 1 | 10 |
| **CF-12** | X | - | - | 1 | - | 2 | 1 | 1 | 2 | 2 | 1 | 10 |
| **CF-13** |  | - | - | 1 | - | 2 | 1 | 1 | 2 | 2 | 1 | 10 |
| **CF-14** |  | - | - | 1 | - | 2 | 1 | 1 | 2 | 2 | 1 | 10 |
| **CF-15** |  | - | - | 1 | - | 2 | 1 | 1 | 2 | 2 | 1 | 10 |
| **CF-16** |  | - | - | 1 | - | 2 | 1 | 1 | 2 | 2 | 1 | 10 |
| **CF-17** |  | - | - | 1 | - | 2 | 1 | 1 | 2 | 2 | 1 | 10 |
| **CF-18** |  | - | - | 1 | - | 2 | 1 | 1 | 2 | 2 | 1 | 10 |
| **CF-19** |  | - | - | 1 | - | 2 | 1 | 1 | 2 | 2 | 1 | 10 |
| **CF-20** |  | - | - | 1 | - | 2 | 1 | 1 | 2 | 2 | 1 | 10 |
| **CF-21** |  | - | - | 1 | - | 2 | 1 | 1 | 2 | 2 | 1 | 10 |
| **CF-22** |  | - | - | 1 | - | 2 | 1 | 1 | 2 | 2 | 1 | 10 |
| **CF-23** |  | - | - | 1 | - | 2 | 1 | 1 | 2 | 2 | 1 | 10 |
| **CF-24** |  | - | - | 1 | - | 2 | 1 | 1 | 2 | 2 | 1 | 10 |
| **CF-25** |  | - | - | 1 | - | 2 | 1 | 1 | 2 | 2 | 1 | 10 |
| **CF-26** |  | - | - | 1 | - | 2 | 1 | 1 | 2 | 2 | 1 | 10 |
| **CF-27** |  | - | - | 1 | - | 2 | 1 | 1 | 2 | 2 | 1 | 10 |
| **CF-28** | X | - | - | 1 | - | 2 | 1 | 1 | 2 | 2 | 1 | 10 |
| **CF-29** |  | - | - | 1 | - | 2 | 1 | 1 | 2 | 2 | 1 | 10 |
| **CF-30** |  | - | - | 1 | - | 2 | 1 | 1 | 2 | 2 | 1 | 10 |
| **CF-31** |  | - | - | 1 | - | 2 | 1 | 1 | 2 | 2 | 1 | 10 |
| **CF-32** |  | - | - | 1 | - | 2 | 1 | 1 | 2 | 2 | 1 | 10 |
| **CF-33** |  | 3 | 2 | - | - | - | 1 | 1 | 1 | 1 | 1 | 10 |
| **CF-34** |  | 3 | 2 | - | - | - | 1 | 1 | 1 | 1 | 1 | 10 |
| **CF-35** | X | - | 3 | - | - | - | 1 | 1 | 2 | 2 | 1 | 10 |
| **CF-36** |  | - | 3 | - | - | - | 1 | 1 | 2 | 2 | 1 | 10 |
| **CF-37** |  | - | - | 1 | - | 2 | 1 | 1 | 2 | 2 | 1 | 10 |
| **CF-38** |  | - | - | 1 | - | 1 | 2 | 1 | 2 | 2 | 1 | 10 |
| **CF-39** | X | - | - | 1 | - | 2 | 1 | 1 | 2 | 2 | 1 | 10 |
| **CF-40** |  | - | - | 1 | - | 2 | 1 | 1 | 2 | 2 | 1 | 10 |
| **CF-41** |  | - | - | 1 | - | 2 | 1 | 1 | 2 | 2 | 1 | 10 |
| **CF-42** |  | - | - | 1 | - | 2 | 1 | 1 | 2 | 2 | 1 | 10 |
| **CF-43** |  | - | - | 1 | - | 2 | 1 | 1 | 2 | 2 | 1 | 10 |
| **CF-44** |  | - | - | 1 | - | 2 | 1 | 1 | 2 | 2 | 1 | 10 |
| **CF-45** |  | - | - | - | 1 | 2 | 1 | 1 | 2 | 2 | 1 | 10 |

**Table S3.** Random Forest cross validation of ten replicates of 45 CF *P. aeruginosa* isolates; isolates which grew mucoid more than once are indicated with a star.

| **Isolate** | **Precision** | **Recall** | **F1 Score** | **Number of Samples** |
| --- | --- | --- | --- | --- |
| **CF_01** | 0.91 | 1.00 | 0.95 | 10 |
| **CF_02*** | 0.64 | 0.90 | 0.75 | 10 |
| **CF_03** | 1.00 | 0.90 | 0.95 | 10 |
| **CF_04** | 0.70 | 0.70 | 0.70 | 10 |
| **CF_05** | 0.00 | 0.00 | 0.00 | 10 |
| **CF_06** | 0.91 | 1.00 | 0.95 | 10 |
| **CF_07** | 0.77 | 1.00 | 0.87 | 10 |
| **CF_08*** | 0.69 | 0.90 | 0.78 | 10 |
| **CF_09** | 0.88 | 0.70 | 0.78 | 10 |
| **CF_10** | 1.00 | 1.00 | 1.00 | 10 |
| **CF_11** | 0.73 | 0.80 | 0.76 | 10 |
| **CF_12*** | 1.00 | 0.60 | 0.75 | 10 |
| **CF_13** | 0.77 | 1.00 | 0.87 | 10 |
| **CF_14** | 1.00 | 1.00 | 1.00 | 10 |
| **CF_15** | 0.91 | 1.00 | 0.95 | 10 |
| **CF_16** | 0.64 | 0.70 | 0.67 | 10 |
| **CF_17** | 1.00 | 1.00 | 1.00 | 10 |
| **CF_18** | 0.00 | 0.00 | 0.00 | 10 |
| **CF_19** | 0.73 | 0.80 | 0.76 | 10 |
| **CF_20** | 0.12 | 0.10 | 0.11 | 10 |
| **CF_21** | 1.00 | 1.00 | 1.00 | 10 |
| **CF_22** | 0.83 | 1.00 | 0.91 | 10 |
| **CF_23** | 1.00 | 1.00 | 1.00 | 10 |
| **CF_24** | 0.88 | 0.70 | 0.78 | 10 |
| **CF_25** | 1.00 | 0.90 | 0.95 | 10 |
| **CF_26** | 0.78 | 0.70 | 0.74 | 10 |
| **CF_27** | 0.82 | 0.90 | 0.86 | 10 |
| **CF_28*** | 0.86 | 0.60 | 0.71 | 10 |
| **CF_29** | 1.00 | 0.80 | 0.89 | 10 |
| **CF_30** | 1.00 | 1.00 | 1.00 | 10 |
| **CF_31** | 1.00 | 0.90 | 0.95 | 10 |
| **CF_32** | 1.00 | 1.00 | 1.00 | 10 |
| **CF_33** | 0.91 | 1.00 | 0.95 | 10 |
| **CF_34** | 0.88 | 0.70 | 0.78 | 10 |
| **CF_35*** | 1.00 | 0.70 | 0.82 | 10 |
| **CF_36** | 1.00 | 0.90 | 0.95 | 10 |
| **CF_37** | 0.80 | 0.80 | 0.80 | 10 |
| **CF_38** | 1.00 | 0.70 | 0.82 | 10 |
| **CF_39*** | 0.82 | 0.90 | 0.86 | 10 |
| **CF_40** | 0.56 | 0.50 | 0.53 | 10 |
| **CF_41** | 1.00 | 1.00 | 1.00 | 10 |
| **CF_42** | 0.73 | 0.80 | 0.76 | 10 |
| **CF_43** | 0.77 | 1.00 | 0.87 | 10 |
| **CF_44** | 0.90 | 0.90 | 0.90 | 10 |
| **CF_45** | 0.82 | 0.9 | 0.86 | 10 |
| **Average/Total** | **0.82** | **0.81** | **0.81** | **450** |

**Table S4.** MLST strain identification of the 45 *P. aeruginosa* isolated from CF patients shown with the results of the one-leave-out cross validation for strain prediction; isolates which grew mucoid more than once are indicated with a star.

| **Strain** | **Number of Isolates** | **Isolate IDs** | **Precision** | **Recall** | **F1 Score** | **Number of Replicates** |
| --- | --- | --- | --- | --- | --- | --- |
| **ST_0009** | 1 | CF_22 | 0.88 | 0.70 | 0.78 | 10 |
| **ST_0014** | 1 | CF_28* | 0.60 | 0.60 | 0.60 | 10 |
| **ST_0017** | 3 | CF_25 ; CF_26 ; CF_42 | 0.91 | 0.97 | 0.94 | 30 |
| **ST_0110** | 1 | CF_04 | 0.67 | 0.60 | 0.63 | 10 |
| **ST_0132** | 1 | CF_19 | 0.89 | 0.80 | 0.84 | 10 |
| **ST_0146** | 1 | CF_07 | 0.77 | 1.00 | 0.87 | 10 |
| **ST_0175** | 1 | CF_27 | 0.91 | 1.00 | 0.95 | 10 |
| **ST_0179** | 2 | CF_14 ; CF_44 | 0.76 | 0.65 | 0.70 | 20 |
| **ST_0195** | 1 | CF_08* | 0.82 | 0.90 | 0.86 | 10 |
| **ST_0217** | 1 | CF_45 | 0.83 | 1.00 | 0.91 | 10 |
| **ST_0235** | 1 | CF_23 | 1.00 | 1.00 | 1.00 | 10 |
| **ST_0244** | 2 | CF_30 ; CF_36 | 1.00 | 1.00 | 1.00 | 20 |
| **ST_0252** | 1 | CF_31 | 1.00 | 0.80 | 0.89 | 10 |
| **ST_0253** | 2 | CF_17 ; CF_32 | 0.90 | 0.90 | 0.90 | 20 |
| **ST_0274** | 1 | CF_40 | 0.89 | 0.80 | 0.84 | 10 |
| **ST_0298** | 2 | CF_10 ; CF_33 | 0.90 | 0.95 | 0.93 | 20 |
| **ST_0381** | 1 | CF_34 | 1.00 | 0.50 | 0.67 | 10 |
| **ST_0390** | 3 | CF_05 ; CF_18 ; CF_20 | 0.76 | 0.93 | 0.84 | 30 |
| **ST_0395** | 2 | CF_01 ; CF_39* | 0.79 | 0.55 | 0.65 | 20 |
| **ST_0560** | 1 | CF_15 | 0.83 | 1.00 | 0.91 | 10 |
| **ST_0612** | 1 | CF_21 | 1.00 | 1.00 | 1.00 | 10 |
| **ST_0633** | 1 | CF_16 | 0.43 | 0.30 | 0.35 | 10 |
| **ST_0954** | 1 | CF_09 | 0.73 | 0.80 | 0.76 | 10 |
| **ST_1207** | 1 | CF_02* | 0.70 | 0.70 | 0.70 | 10 |
| **ST_1225** | 1 | CF_43 | 0.91 | 1.00 | 0.95 | 10 |
| **ST_1248** | 1 | CF_29 | 0.89 | 0.80 | 0.84 | 10 |
| **ST_1641** | 2 | CF_12* ; CF_35* | 0.55 | 0.85 | 0.67 | 20 |
| **ST_2362** | 1 | CF_11 | 0.71 | 0.50 | 0.59 | 10 |
| **ST_2859** | 1 | CF_24 | 1.00 | 0.90 | 0.95 | 10 |
| **ST_2951** | 1 | CF_13 | 1.00 | 1.00 | 1.00 | 10 |
| **ST_3016** | 1 | CF_41 | 1.00 | 1.00 | 1.00 | 10 |
| **ST_3017** | 1 | CF_06 | 0.91 | 1.00 | 0.95 | 10 |
| **ST_3018** | 1 | CF_03 | 0.90 | 0.90 | 0.90 | 10 |
| **ST_3019** | 1 | CF_38 | 0.80 | 0.80 | 0.80 | 10 |
| **ST_3020** | 1 | CF_37 | 1.00 | 0.60 | 0.75 | 10 |
| **Average/Total** | **45** | **-** | **0.84** | **0.83** | **0.83** | **450** |
